# Patients with progression of spinal metastases who present to the clinic have better outcomes compared to those who present to the emergency department

**DOI:** 10.1002/cam4.6601

**Published:** 2023-09-30

**Authors:** Joseph R. Linzey, Varun G. Kathawate, Michael J. Strong, Kayla Roche, Peyton E. Goethe, Lila R. Tudrick, Johan Lee, Arushi Tripathy, Sravanthi Koduri, Ayobami L. Ward, Oludotun Ogunsola, Mark M. Zaki, Rushikesh S. Joshi, Grant Weyburne, Charles S. Mayo, Joseph R. Evans, William C. Jackson, Nicholas J. Szerlip

**Affiliations:** ^1^ Department of Neurosurgery University of Michigan Ann Arbor Michigan USA; ^2^ School of Medicine University of Michigan Ann Arbor Michigan USA; ^3^ Department of Radiation Oncology University of Michigan Ann Arbor Michigan USA

**Keywords:** acute care, neurologic complications, outcomes, spinal metastases, spine oncology clinic, stereotactic body radiation therapy

## Abstract

**Background:**

As cancer therapies have improved, spinal metastases are increasingly common. Resulting complications have a significant impact on patient's quality of life. Optimal methods of surveillance and avoidance of neurologic deficits are understudied. This study compares the clinical course of patients who initially presented to the emergency department (ED) versus a multidisciplinary spine oncology clinic and who underwent stereotactic body radiation therapy (SBRT) secondary to progression/presentation of metastatic spine disease.

**Methods:**

We performed a retrospective analysis of a prospectively maintained database of adult oncologic patients who underwent spinal SBRT at a single hospital from 2010 to 2021. Descriptive statistics and survival analyses were performed.

**Results:**

We identified 498 spinal radiographic treatment sites in 390 patients. Of these patients, 118 (30.3%) presented to the ED. Patients presenting to the ED compared to the clinic had significantly more severe spinal compression (52.5% vs. 11.7%; *p* < 0.0001), severe pain (28.8% vs. 10.3%; *p* < 0.0001), weakness (24.5% vs. 4.5%; *p* < 0.0001), and difficulty walking (24.5% vs. 4.5%; *p* < 0.0001). Patients who presented to the ED compared to the clinic were significantly more likely to have surgical intervention followed by SBRT (55.4% vs. 15.3%; *p* < 0.0001) compared to SBRT alone. Patients who presented to the ED compared to the clinic had a significantly quicker interval to distant spine progression (5.1 ± 6.5 vs. 9.1 ± 10.2 months; *p* = 0.004), systemic progression (5.1 ± 7.2 vs. 9.2 ± 10.7 months; *p* < 0.0001), and worse overall survival (9.3 ± 10.0 vs. 14.3 ± 13.7 months; *p* = 0.002).

**Conclusion:**

The establishment of multidisciplinary spine oncology clinics is an opportunity to potentially allow for earlier, more data‐driven treatment of their spinal metastatic disease.

## 1 INTRODUCTION

Despite stability in the annual incidence of new cancer diagnoses, cancer death rates have declined over the past several years by virtue of advances in screening and treatment.^1^ However, as patients with cancer are living longer, spinal metastases are becoming increasingly more common, occurring in approximately 15% of patients with solid tumors.^2^, ^3^ Spinal metastases are particularly burdensome, often causing substantial pain, neurologic dysfunction, and decreased performance status.^4^, ^5^, ^6^, ^7^ They present a considerable risk for spinal cord compression, which can result in permanently disabling oncologic emergencies.^8^ Approximately 10% of patients with spinal metastases develop spinal cord compression, and there is frequently a significant delay between the onset of symptoms and diagnosis.^3^, ^9^ These complications can have a significant impact on patients' quality of life.^10^, ^11^

A few solutions have been explored to optimize disease surveillance and improve outcomes for patients with spinal metastases. One solution is the establishment of multidisciplinary clinics that leverage radiotherapeutic, surgical, and interventional approaches.^12^ Previous work has suggested that planned, elective care—consistent with the multidisciplinary clinic model—may be more resource‐effective and prolong patient survival when compared to care provided in the emergency setting.^13^ However, the outcomes and disease trajectories of patients initially presenting to a multidisciplinary clinic versus an emergency department (ED) have not been adequately explored.

The purpose of this study is to compare the clinical courses of patients who initially presented to the ED versus initially presented to a multidisciplinary spinal oncology clinic with progression of spinal metastases before undergoing stereotactic body radiation therapy (SBRT) with or without surgical intervention.

## 2 METHODS

### 2.1 Study design and patient selection

We performed a retrospective analysis of a prospectively maintained database of consecutive adult oncologic patients who underwent SBRT to the spine for metastatic disease at a single, large tertiary care facility from 2010 to 2021. Data were collected using our custom system, the Michigan Radiation Oncology Analytics Resource (MROAR), which aggregates, integrates, and harmonizes data from the electronic systems used to treat patients, including our electronic record system (EPIC), radiation oncology information system (ARIA, Varian Medical Systems), and treatment planning system (Eclipse, Varian Medical Systems).^14^ All included patients underwent SBRT based on our established treatment algorithm.^12^ It was determined that they had a sufficiently high functional status, were appropriate candidates for SBRT therapy, and often had systemic treatment options remaining. Patients with poor performance status or advanced disease with limited treatment options remaining did not receive SBRT and were thus not included in this dataset. Adult patients (≥18 years old) were included. Approval for this study was obtained from our local Institutional Review Board.

### 2.2 Clinical data

Primary outcomes for this study were progression of the tumor (local or in‐field, distant spine, and systemic), which was defined as radiographic progression on follow‐up imaging as well as by a clinical consensus between the treating physicians. Secondary outcomes included degree of pain at presentation and at 1 and 3 months post‐SBRT, ambulation status at presentation and at 1 and 3 months post‐SBRT, and motor deficits at presentation and at 1 and 3 months post‐SBRT.

Demographic data were prospectively entered for each patient as the patient began SBRT. Variables included age at treatment, sex, body mass index, race (White, African American, Asian, other, and unknown), marital status (single, married, divorced, widowed, and unknown), insurance type (private, Medicare, Medicaid, uninsured, and unknown), and whether the patient had a primary care physician. Other variables which were prospectively maintained were whether or not the patient underwent surgery (transpedicular decompression with fusion; vertebroplasty on its own was not considered surgery for this study), the histology of the tumor, whether there were contiguous spinal levels of disease, whether the patient had previously undergone radiation therapy at the level of interest, and the dose of radiation which was converted to biologically effective doses for standardization.

Additional variables were retrospectively gathered from this prospective cohort, including: initial presentation to the ED or the clinic, the Bilsky score of the lesion at the level of worst compression; in‐field progression of cancer and date of in‐field progression; distant spine progression and date of distant spine progression; systemic progression and date of systemic progression; degree of pain (no pain, mild pain [1–3/10], moderate pain [4–6/10], severe pain [7–10/10]) at presentation and at 1 and 3 months post‐SBRT; ambulation status (ambulatory, difficulty walking, wheelchair bound) at presentation and at 1 and 3 months post‐SBRT; and motor deficits (no deficits, weakness, myelopathy, both weakness and myelopathy) at presentation and at 1 and 3 months post‐SBRT.

Of note, the Bilsky scores were grouped into minimal compression (0, 1a, 1b, and 1c), severe compression (2 or 3), and no data (no MRI available before treatment began or sacral lesion).

### 2.3 Clinical decision making and follow‐up in a multidisciplinary spinal oncology clinic

Patients with metastatic spinal tumors who present to the ED or to multidisciplinary clinic are initially evaluated according to our published algorithm, which has been discussed previously.^12^ If the patient needs surgery for mechanical stabilization or for neurologic protection, they undergo surgery. This decision is paired with the decision of utilizing either fractionated external beam or high‐dose SBRT. If a patient is determined to be a good candidate for SBRT therapy, a discussion is conducted between the neurosurgery and radiation oncology teams regarding whether separation surgery is necessary to obtain sufficiently high doses of SBRT. If it is determined that surgical separation is needed, the patient undergoes transpedicular decompression at the level of the tumor with circumferential decompression of the thecal sac with separation that is assessed intraoperatively by ultrasound. Once the transpedicular decompression is performed, we place screws two levels above and below the decompression.

Patients who underwent SBRT were followed in a multidisciplinary spinal oncology clinic where their care was coordinated between their neurosurgical team, radiation oncologists, medical oncologists, physical therapists, and other ancillary teams. Patients were seen in the clinic at 1 and 3 months for examination and assessment of treatment effects as well as every 3–6 months for a surveillance total spine MRI. The need for additional treatment was determined in the multidisciplinary clinic.

### 2.4 Statistical analysis

We examined the association of each variable against initial presentation to the ED versus the clinic using the chi‐squared test, Fisher exact test, or *t*‐test, depending on the sample size and whether the variable was continuous or categorical. Continuous variables are presented as mean with standard deviations. Survival analyses were utilized to analyze the associations between in‐field progression and systemic progression against presentation to the ED versus the clinic. We performed sensitivity analyses for these associations by splitting the cohort into different histologies to determine if a specific pathology was driving the in‐field progression or systemic progression. We performed a sub‐analysis of the data, re‐running these same statistical tests while only looking at the patients who presented to the ED. A two‐sided *p* ≤ 0.05 was considered to be statistically significant. All data were analyzed using SAS 9.4 software (SAS Institute Inc.).

## 3 RESULTS

### 3.1 Total cohort

A total of 498 radiographic treatment sites treated with SBRT in 390 patients were identified. Of the patients, 65.4% were male with an average age of 62.6 ± 12.6 years. Of these 390 patients, 118 (30.3%) initially presented to the ED and 272 (69.7%) initially presented to the multidisciplinary spinal oncology clinic. Demographic data are summarized in Table 1. The six most common treatment center histologies of patients undergoing SBRT were prostate cancer, renal cell carcinoma, non‐small cell lung cancer, sarcoma, breast cancer, and melanoma (Table 2). Patients with prostate cancer were significantly more likely to initially present to the clinic (24.8 vs. 8.6%), while patients with melanoma were significantly more likely to present to the ED initially (10.8% vs. 2.8%, *p* < 0.0001; Table 2). Patients presenting to ED had significantly more cervical disease compared to patients presenting to the clinic (16.6% vs. 9.5%; *p* = 0.02). There were no significant differences between patients presenting with thoracic or lumbar disease.

**TABLE 1:** Demographic information of patients presenting to the ED compared to the clinic.

	Presenting to the ED *n* = 118	Presenting to the clinic *n* = 272	*p*
Age (years)	61.1 (13.0)	63.2 (12.4)	0.13
Gender			
Female	47 (39.8%)	88 (32.4%)	0.15
Body‐mass index	27.6 (8.1)	27.6 (5.3)	0.96
Race			
White	99 (83.9%)	241 (88.6%)	0.25
Black	12 (10.2%)	13 (4.8%)	
Asian	1 (0.9%)	6 (2.2%)	
Other	2 (1.7%)	6 (2.2%)	
Unknown	4 (3.4%)	6 (2.2%)	
Marital status			
Married	70 (59.3%)	173 (63.6%)	0.83
Divorced	6 (5.1%)	15 (5.5%)	
Single	14 (11.9%)	30 (11.0%)	
Widowed	3 (2.5%)	9 (3.3%)	
Unknown	25 (21.2%)	45 (16.5%)	
Insurance type			
Private	66 (55.9%)	164 (60.3%)	0.01
Medicare	39 (33.1%)	97 (35.7%)	
Medicaid	4 (3.4%)	0 (0.0%)	
Uninsured	6 (5.1%)	9 (3.3%)	
Unknown	3 (2.5%)	2 (0.7%)	
The presence of primary care physician			
No	8 (6.8%)	14 (5.2%)	0.27
Yes	107 (90.7%)	256 (94.1%)	
Unknown	3 (2.5%)	2 (0.7%)	
Surgical intervention			
No	53 (44.9%)	232 (85.3%)	<0.0001
Yes	65 (55.1%)	40 (14.7%)	

Abbreviation: ED, emergency department.

**TABLE 2:** Demographic information of patients' tumor treatment centers.

	Presenting to the ED *n* = 139	Presenting to the clinic *n* = 359	*p*
Histology categories			
Prostate cancer	12 (8.6%)	89 (24.8%)	<0.0001
Renal cell carcinoma	23 (16.6%)	54 (15.0%)	
Non‐small cell lung cancer	25 (18.0%)	46 (12.8%)	
Sarcoma	12 (8.6%)	30 (8.4%)	
Breast cancer	7 (5.0%)	29 (8.1%)	
Melanoma	15 (10.8%)	10 (2.8%)	
Thyroid cancer	0 (0.0%)	18 (5.0%)	
Bladder cancer	3 (2.2%)	13 (3.6%)	
Liver cancer	3 (2.2%)	10 (2.8%)	
Oropharyngeal cancer	6 (4.3%)	7 (2.0%)	
Colorectal cancer	3 (2.2%)	8 (2.2%)	
Neuroendocrine tumor	4 (2.9%)	6 (1.7%)	
Pancreatic cancer	4 (2.9%)	5 (1.4%)	
Esophageal cancer	1 (0.7%)	8 (2.2%)	
Blood vessel tumors	3 (2.2%)	5 (1.4%)	
Salivary cancer	1 (0.7%)	6 (1.7%)	
Primary bone tumor	1 (0.7%)	4 (1.1%)	
Adrenal cancer	3 (2.2%)	0 (0.0%)	
Radiation sensitivity of tumor			
Sensitive	22 (15.8%)	119 (33.2%)	0.0005
Intermediate	55 (39.6%)	122 (34.0%)	
Resistant	62 (44.6%)	118 (32.9%)	
Tumor location			
Cervical spine	23 (16.6%)	34 (9.5%)	0.02
Thoracic spine	83 (59.7%)	208 (57.9%)	
Lumbar spine	32 (23.0%)	99 (27.6%)	
Sacrum	1 (0.7%)	18 (5.0%)	
Bilsky score at presentation grouped			
Minimal compression (Bilsky 0, 1a, 1b, 1c)	63 (45.3%)	248 (69.1%)	<0.0001
Severe compression (Bilsky 2 or 3)	73 (52.5%)	42 (11.7%)	
Other (no MRI or sacrum)	3 (2.2%)	69 (19.2%)	
Contiguous spinal levels involved	77 (55.4%)	120 (33.4%)	<0.0001
Prior radiation therapy to site	19 (13.7%)	41 (11.4%)	0.49
Biologically effective dose (Gy)	54.1 (23.6)	52.9 (10.2)	0.54

Abbreviation: ED, emergency department.

Patients presenting to ED had significantly higher‐grade compression compared to patients who had spine disease identified in clinic (Bilsky grade 2 or 3; 52.5% vs. 11.7%; *p* < 0.0001; Table 2).

**FIGURE 1:**
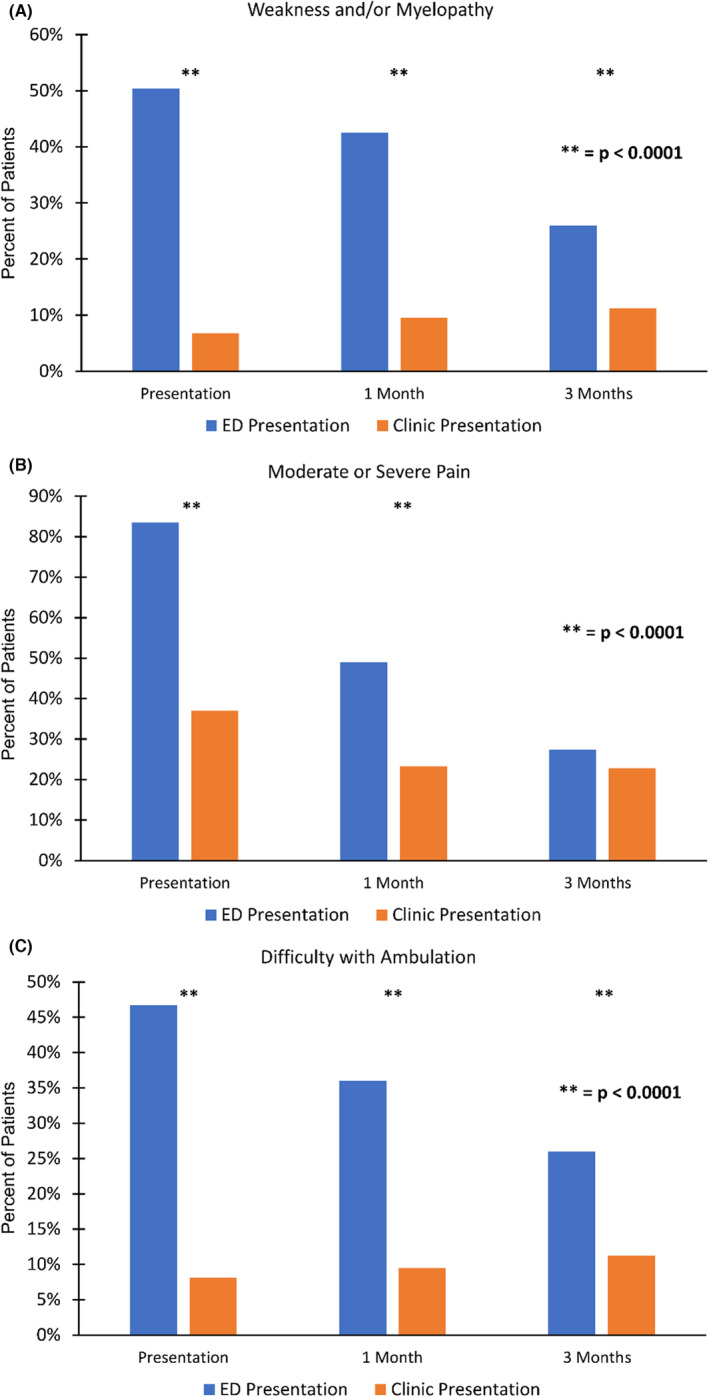
Symptoms of patients who initially presented to the emergency department (ED) versus a multidisciplinary spinal oncology clinic at presentation, 1 month after stereotactic body radiation therapy (SBRT) therapy, and 3 months after SBRT therapy. (A) Weakness and/or myelopathy was significantly worse in patients who presented to the ED compared to the clinic at presentation, 1 month after SBRT, and 3 months after SBRT. (B) Moderate or severe pain was significantly worse in patients who presented to the ED compared to the clinic at presentation and at 1 month after SBRT. However, by 3 months after SBRT, there was no difference in moderate/severe pain between the groups. (C) Difficulty with ambulation was significantly worse in patients who presented to the ED compared to the clinic at presentation, 1 month after SBRT, and 3 months after SBRT.

Patients presenting to ED had more severe symptoms. They presented with a higher incidence of severe pain (7–10/10; 28.8% vs. 10.3%; *p* < 0.0001), weakness (24.5% vs. 4.5%, *p* < 0.0001), and difficulty walking (24.5% vs. 4.5%, *p* < 0.0001) compared to patients who initially presented to the clinic (Table S1; Figure 1). Patients who initially presented to the ED continued to have worse weakness and difficulty ambulating at 1 and 3 months following SBRT compared to patients presenting to the clinic (Figure 1A,C). While patients who presented to the ED had worse pain than patients who presented to the clinic at 1 month, that difference was no longer present by 3 months post‐SBRT (Figure 1B).

There was a significantly higher rate of surgery in patients presenting to the ED. Given the high level of compression and the worse pain, weakness, and ambulation status at presentation, patients who presented to the ED compared to the clinic were significantly more likely to have surgical intervention before receiving SBRT (55.1% vs. 14.7%, *p* < 0.0001; Table 1).

**FIGURE 2:**
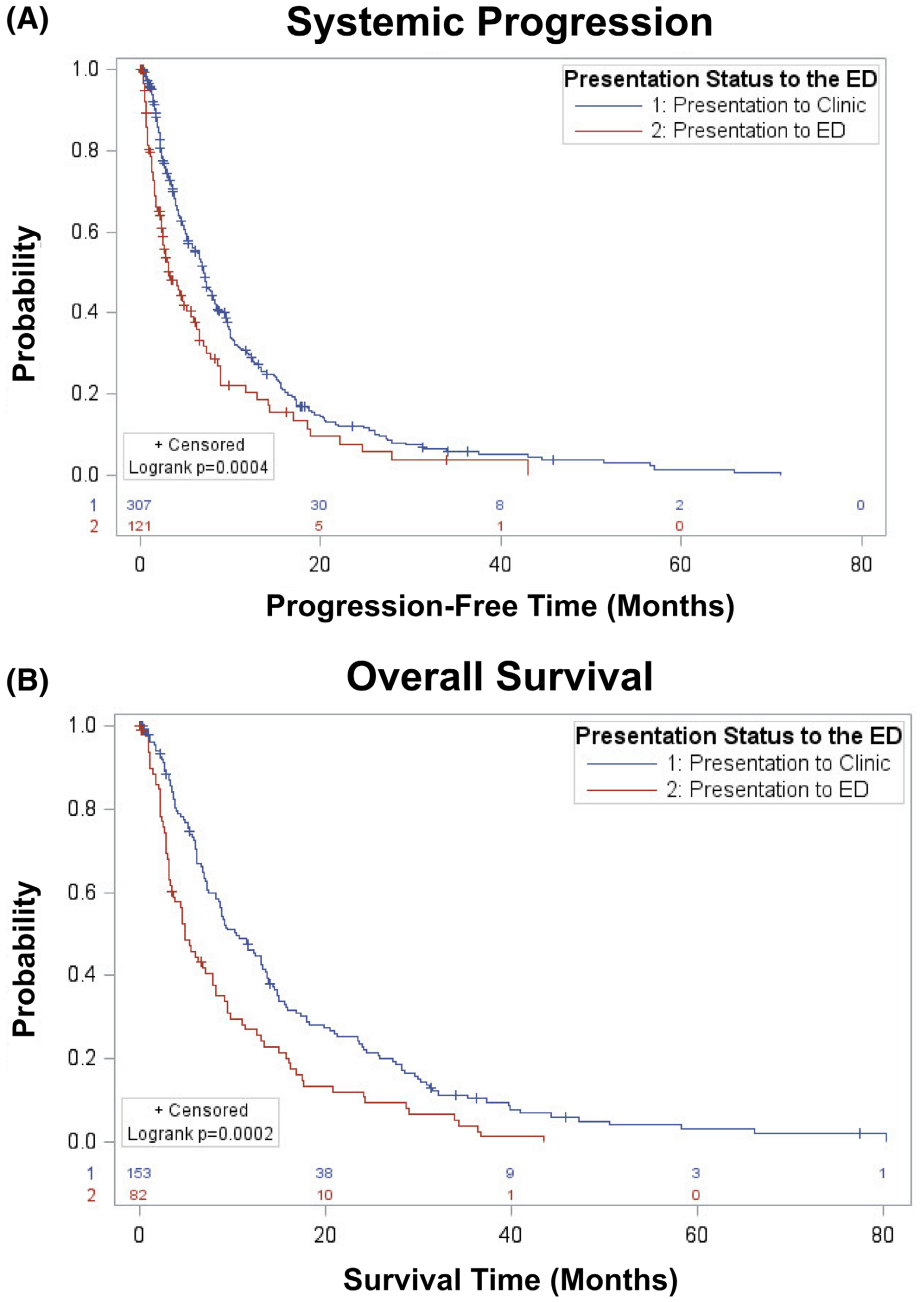
(A) Patients who presented to the clinic had significantly longer systemic progression‐free time in months compared to patients who presented to the emergency department (ED). (B) Patients who presented to the clinic had significantly longer overall survival time in months compared to patients who presented to the ED.

The ED group had a faster time to progression of new spine metastases. Patients who presented to the ED compared to the clinic did not have statistically significant differences in the occurrences of in‐field or distant spinal progression. All patients had a progression rate of distant spinal metastases of about 35% (Table S1). However, distal spinal progression occurred significantly quicker in patients who presented to the ED compared to the clinic (5.1 ± 6.5 vs. 9.1 ± 10.2 months, *p* = 0.004; Table S1). Additionally, overall survival was 14.3 ± 13.7 months for clinic patients compared to 9.3 ± 10.0 months for patients presenting to the ED (*p* = 0.002; Table S1). Patients who presented to the ED initially did have significantly higher rates of systemic progression and decreased overall survival (Figure 2). Patients also had faster time to systemic progression if they presented to the ED compared to the clinic (5.1 ± 7.2 vs. 9.2 ± 10.7 months, *p* < 0.0001). To ensure these differences in rates of systemic progression were not driven by prostate cancer, which was more likely to present to the clinic and to have slower rates of systemic progression, a survival plot was performed without any of the prostate cancer treatment sites as a sensitivity analysis. The patients who presented to the ED continued to have worse rates of systemic progression compared to the clinic patients, suggesting that this finding is not driven by specific histologies (*p* = 0.03).

### 3.2 Sub‐analysis: Patients who presented to the ED only

**FIGURE 3:**
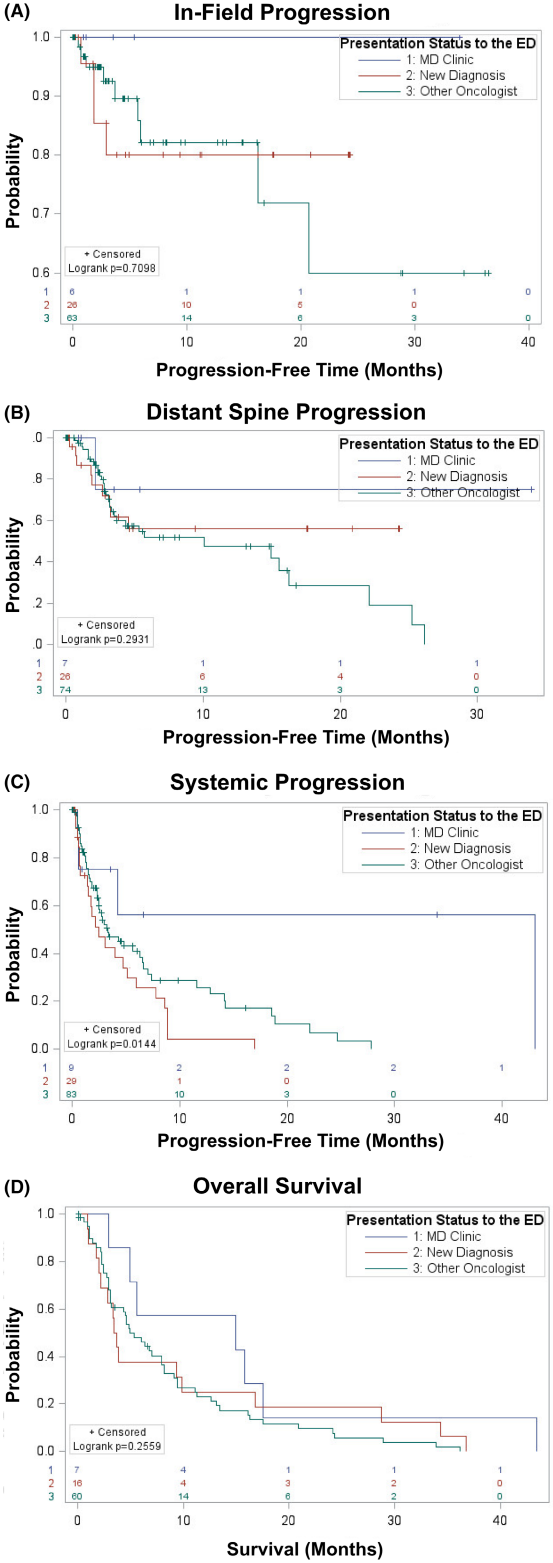
Survival plots for (A) in‐field progression, (B) distant spine progression, (C) systemic progression, and (D) overall survival among patients who presented to a multidisciplinary spinal oncology clinic, got a new oncologic diagnosis in the emergency department (ED), or were followed up by a different oncologist. There was a statistically significant difference in systemic progression (C) among patients who presented to multidisciplinary spinal oncology clinic, got a new oncologic diagnosis in the ED, or were followed by a different oncologist. Patients who presented to multidisciplinary spinal oncology clinic had significantly longer progression‐free time compared to the other two groups.

In the 118 patients who presented to the ED, there were 139 treatment sites. The majority of patients who presented to the ED were followed by an oncologist outside of the multidisciplinary spinal oncology clinic (71.1%) compared to 21.1% with a new oncologic diagnosis, and only 7.6% were previously seen in multidisciplinary spinal oncology clinic. Patients who were followed in multidisciplinary spinal oncology clinic were significantly more likely to undergo surgical intervention in addition to SBRT compared to patients with a new oncologic diagnosis or who were followed by other oncologists (80.0% vs. 67.6% vs. 48.4%, *p* = 0.04). No patients who were followed in multidisciplinary spinal oncology clinic had in‐field progression. While there was no statistically significant difference for in‐field progression, distant spinal progression, or overall survival among these three groups, patients who were followed in multidisciplinary spinal oncology clinic did have significantly longer systemic progression‐free time (Figure 3; Table 3).

**TABLE 3:** Progression and survival of patients who present to the emergency department.

	New cancer diagnosis *n* = 25	Followed in multidisiplinary clinic *n* = 9	Followed by a different oncologist *n* = 84
In‐field progression			
Mean (months)	1.8 (0.9)	‐	6.4 (7.2)
Median (months)	1.85	‐	3.7
Distant spinal progression			
Mean (months)	2.1 (1.4)	2.2 (−)	6.0 (7.2)
Median (months)	1.9	2.2	2.9
Systemic progression			
Mean (months)	4.1 (4.1)	12.1 (20.7)	5.0 (6.6)
Median (months)	2.4	2.4	2.4
Overall survival			
Mean (months)	10.1 (12.3)	15.0 (13.9)	8.3 (8.5)
Median (months)	3.6	14.9	4.9

## 4 DISCUSSION

As cancer death rates have continued to decline, spinal metastases are becoming increasingly more common, occurring in approximately 15% of patients.^1^, ^2^, ^3^ Spinal metastases can cause significant pain, neurological dysfunction, and decreased performance status, which can have a significant impact on patients' quality of life.^4^, ^5^, ^6^, ^7^ Given the increased risk of spinal metastases, numerous institutions have established multidisciplinary clinics that bring together neurosurgery, radiation oncology, and medical oncology services to provide care that is more resource efficient and comprehensive.^12^ Unfortunately, nearly a third of patients in our population still discover that they have spinal metastases by presenting to the ED rather than becoming integrated into the multidisciplinary spinal oncology clinic by referral from their primary oncologists. Patients who presented to the ED with a spinal event instead of the clinic had significantly worse epidural compression, pain, weakness, and difficulty with ambulation, and were significantly quicker to have distant spine progression and systemic progression.

Patients who initially presented to the ED with a spinal event had higher degrees of radiographic compression and more severe symptoms. While it is logical that patients with worse clinical symptoms would have greater degrees of compression and would thus present to the ED, optimal care for these patients continues to come from a multidisciplinary team that can discuss ideal treatment regimens that are most in line with a patient's goals of care.^12^ Zanaty and George^13^ found that in their patient population, about 75% of patients with spinal metastases who presented to the ED were emergently admitted, whereas the remaining 25% were planned, elective admissions. Of the patients who were admitted emergently, almost half did not undergo surgery during that admission and the cost of the emergent care was significantly higher than the cost of planned admissions.^13^ Similarly, de la Garza Ramos et al.^15^ demonstrated that patients with metastatic spinal disease who were transferred between hospitals had significantly more severe clinical presentations and higher rates of inpatient complications compared to patients directly admitted through clinics. Both studies support the idea that care managed through a multidisciplinary spinal oncology clinic, which coordinates and streamlines care, can provide improved and less costly care for patients and hospital systems.

Given the higher rate of symptomatic compression in patients who initially presented to the ED instead of a multidisciplinary spinal oncology clinic, significantly more of those patients underwent surgery prior to receiving SBRT.^16^ When a patient presents to the ED, there is sometimes an increase in pressure to treat the patient immediately given the acuity of the patient presentation and expectation for urgent management. Early referral and presentation to a multidisciplinary spinal oncology clinic can allow for alignment of a treatment paradigm with the patient's goals of care. Thoughtful discussion is often needed to fully assess a patient's performance status, systemic burden of disease, and systemic treatment options to allow for the best treatment course.^12^, ^17^

Patients who presented to the ED were significantly quicker to develop distant spinal progression and systemic progression. We performed sensitivity analyses to ensure that this finding was not due to specific disease histologies and found that it was not. When patients present with spine progression to a multidisciplinary clinic, their systemic treatment is usually optimized around their spine treatment. Although we did not have the ability to examine this, we theorize that the length of time off systemic treatment was shorter for the patients who presented to the clinic. In addition, a subset of the patients who presented to the ED were not under the care of a primary oncologist, which may have led to delays in systemic treatments. This points to the fact that early presentation to a multidisciplinary spinal oncology clinic can identify clinical changes at an earlier time point and provide treatments on the basis of diagnostic findings to prolong progression‐free survival. Earlier identification of progression can lead to improved care and more timely surgery, if needed, which has improved outcomes and is more cost effective than delayed surgery.^17^, ^18^

Our sub‐analysis demonstrated that only about 7% of the patients who presented to the ED were followed in a multidisciplinary spinal oncology clinic. Even among the small number of patients in this group, we demonstrated significantly longer systemic progression‐free time. We postulate that this is likely due to the improved coordination that is provided by a multidisciplinary clinic, which allows for cohesive care without prolonged delays in the administration of systemic therapy options. Progression is quickly detected and since all of the treatment teams are already involved in the patient's care, the optimal therapy can be administered. Additional prospective studies with larger cohorts need to be performed to further elucidate the benefits of the coordinated care provided by multidisciplinary spinal oncology clinics.

### 4.1 Limitations

While this is a prospectively maintained database, many of the variables we utilized in this study were retrospectively gathered, which introduces potential biases into the data. Future prospective trials will significantly improve our understanding of the benefits of multidisciplinary spinal oncology clinics for patients. Additionally, we do not have granular detail regarding reasons why patients initially presented to the ED rather than a multidisciplinary spinal oncology clinic. We were unable to determine causes for why some patients were referred to the multidisciplinary spinal oncology clinic and some patients remained solely with their primary oncologist. Additionally, laboratory values such as pre‐albumin were not included in this analysis. Laboratory values can be important predictors of survival and frailty.^19^ In future work, prospectively collected data will include these laboratory values.

## 5 CONCLUSION

Patients with metastatic spinal disease progression who present to the ED and who are deemed fit for aggressive therapy by consensus guidelines have poorer survival, faster time to progression to other spine sites, and faster time to systemic progression than those who present to a multidisciplinary spinal oncology clinic, despite having similar prognoses at the time of treatment. The establishment of multidisciplinary spinal oncology clinics provides an opportunity to follow patients, quickly identify spinal progression, and potentially allow for earlier, more coordinated and data‐driven treatment of the spinal metastatic disease.

## AUTHOR CONTRIBUTIONS

**Joseph R. Linzey:** Data curation (equal); formal analysis (lead); investigation (equal); methodology (supporting); software (lead); validation (supporting); visualization (lead); writing – original draft (lead); writing – review and editing (equal). **Varun G. Kathawate:** Data curation (supporting); formal analysis (supporting); investigation (equal); methodology (supporting); validation (supporting); visualization (supporting); writing – original draft (supporting); writing – review and editing (supporting). **Michael J. Strong:** Data curation (equal); formal analysis (supporting); investigation (equal); methodology (supporting); validation (supporting); visualization (supporting); writing – review and editing (supporting). **Kayla Roche:** Investigation (equal); writing – original draft (supporting); writing – review and editing (supporting). **Peyton E. Goethe:** Data curation (equal); investigation (equal); writing – review and editing (supporting). **Lila R. Tudrick:** Data curation (equal); investigation (equal); writing – review and editing (supporting). **Johan Lee:** Data curation (equal); investigation (equal); writing – review and editing (supporting). **Arushi Tripathy:** Data curation (equal); investigation (equal); writing – review and editing (supporting). **Sravanthi Koduri:** Data curation (equal); investigation (equal); writing – review and editing (supporting). **Ayobami L. Ward:** Data curation (equal); investigation (equal); writing – review and editing (supporting). **Oludotun Ogunsola:** Data curation (equal); investigation (equal); writing – review and editing (supporting). **Mark M. Zaki:** Data curation (equal); investigation (equal); writing – review and editing (supporting). **Rushikesh S. Joshi:** Data curation (equal); investigation (equal); writing – review and editing (supporting). **Grant Weyburne:** Conceptualization (supporting); formal analysis (supporting); investigation (equal); methodology (supporting); supervision (supporting); validation (supporting); visualization (supporting); writing – original draft (supporting); writing – review and editing (equal). **Charles S. Mayo:** Conceptualization (supporting); formal analysis (supporting); investigation (equal); methodology (supporting); supervision (supporting); validation (supporting); visualization (supporting); writing – original draft (supporting); writing – review and editing (equal). **Joseph R. Evans:** Conceptualization (supporting); formal analysis (supporting); investigation (equal); methodology (supporting); supervision (supporting); validation (supporting); visualization (supporting); writing – original draft (supporting); writing – review and editing (equal). **William C. Jackson:** Conceptualization (supporting); formal analysis (supporting); investigation (equal); methodology (supporting); supervision (supporting); validation (supporting); visualization (supporting); writing – original draft (supporting); writing – review and editing (equal). **Nicholas J. Szerlip:** Conceptualization (lead); formal analysis (supporting); investigation (equal); methodology (lead); supervision (lead); validation (lead); visualization (supporting); writing – original draft (supporting); writing – review and editing (equal).

## FUNDING INFORMATION

This study received no external funding.

## CONFLICT OF INTEREST STATEMENT

The authors have no conflicts of interest.

## ETHICAL APPROVAL

University of Michigan Institutional Review Board.

## Supporting information


Table S1.

## Data Availability

Research data are not shared.
