# Supplementary material for: Patients with progression of spinal metastases who present to the clinic have better outcomes compared to those who present to the emergency department
Source: Cancer Med. 2023 Sep 30;12(19):20177–87. doi: 10.1002/cam4.6601 (PMC10587959; doi:10.1002/cam4.6601)
Supplement: Supplementary file 1 — Table S1. [file CAM4-12-20177-s001.docx]

**SUPPLEMENTAL TABLE** Outcomes for treatment centers

|  | **Presenting to the ED n = 139** | **Presenting to the clinic n = 359** | ***p*** |
| --- | --- | --- | --- |
| In-field progression post-treatment |  |  | .56 |
| No | 44 (31.7%) | 135 (37.6%) |  |
| Yes | 13 (9.4%) | 31 (8.6%) |  |
| Deceased/hospice before follow-up | 77 (55.4%) | 185 (51.5%) |  |
| Unknown | 5 (3.6%) | 8 (2.2%) |  |
| Time to in-field progression (months) | 5.0 (6.3) | 8.9 (6.4) | .07 |
| Distant spine progression post-treatment |  |  | .05 |
| No | 32 (23.0%) | 111 (30.9%) |  |
| Yes | 43 (30.9%) | 130 (36.2%) |  |
| Deceased/hospice before follow-up | 59 (42.5%) | 111 (30.9%) |  |
| Unknown | 5 (3.6%) | 7 (2.0%) |  |
| Time to distant spine progression (months) | 5.1 (6.5) | 9.1 (10.2) | .004 |
| Systemic progression post-treatment |  |  | .02 |
| No | 18 (13.0%) | 52 (14.5%) |  |
| Yes | 83 (59.7%) | 250 (69.6%) |  |
| Deceased/hospice before follow-up | 31 (22.3%) | 41 (11.4%) |  |
| Unknown | 7 (5.0%) | 16 (4.5%) |  |
| Time to systemic progression (months) | 5.1 (7.2) | 9.2 (10.7) | <.0001 |
| Overall survival (months) | 9.3 (10.0) | 14.3 (13.7) | .002 |
| Pain present on presentation |  |  | <.0001 |
| None | 8 (5.8%) | 105 (29.3%) |  |
| Mild (1-3) | 15 (10.8%) | 121 (33.7%) |  |
| Moderate (4-6) | 76 (54.7%) | 96 (26.7%) |  |
| Severe (7-10) | 40 (28.8%) | 37 (10.3%) |  |
| Ambulation status at presentation |  |  | <.0001 |
| Ambulatory | 74 (53.2%) | 330 (92.9%) |  |
| Difficulty (cane/walker) | 63 (45.3%) | 29 (8.1%) |  |
| Wheelchair | 2 (1.4%) | 0 (0.0%) |  |
| Motor deficits at presentation |  |  | <.0001 |
| No deficits | 69 (49.6%) | 335 (93.3%) |  |
| Weakness | 34 (24.5%) | 16 (4.5%) |  |
| Myelopathy | 12 (8.6%) | 6 (1.7%) |  |
| Both weakness and myelopathy | 24 (17.3%) | 2 (0.6%) |  |
| Pain present at 1 month post-treatment |  |  | <.0001 |
| None | 18 (13.0%) | 105 (29.3%) |  |
| Mild (1-3) | 32 (23.0%) | 102 (28.4%) |  |
| Moderate (4-6) | 44 (31.7%) | 63 (17.6%) |  |
| Severe (7-10) | 24 (17.3%) | 20 (5.6%) |  |
| No data at time point | 21 (15.1%) | 69 (19.2%) |  |
| Ambulation status at 1 month post-treatment |  |  | <.0001 |
| Ambulatory | 66 (47.5%) | 264 (73.5%) |  |
| Difficulty (cane/walker) | 44 (31.7%) | 25 (7.0%) |  |
| Wheelchair | 6 (4.3%) | 1 (0.3%) |  |
| No data at time point | 23 (16.6%) | 69 (19.2%) |  |
| Motor deficits at 1 month post-treatment |  |  | <.0001 |
| No deficits | 57 (41.0%) | 255 (71.0%) |  |
| Weakness | 39 (28.1%) | 29 (8.1%) |  |
| Myelopathy | 2 (1.4%) | 2 (0.6%) |  |
| Both weakness and myelopathy | 18 (13.0%) | 3 (0.8%) |  |
| No data at time point | 23 (16.6%) | 70 (19.5%) |  |
| Pain present at 3 months post-treatment |  |  | <.0001 |
| None | 20 (14.4%) | 110 (30.6%) |  |
| Mild (1-3) | 28 (20.1%) | 91 (25.4%) |  |
| Moderate (4-6) | 23 (16.6%) | 60 (16.7%) |  |
| Severe (7-10) | 15 (10.8%) | 22 (6.1%) |  |
| No data at time point | 53 (38.1%) | 76 (21.2%) |  |
| Ambulation status at 3 months post-treatment |  |  | <.0001 |
| Ambulatory | 46 (33.1%) | 247 (68.8%) |  |
| Difficulty (cane/walker) | 30 (21.6%) | 29 (8.1%) |  |
| Wheelchair | 6 (4.3%) | 6 (1.7%) |  |
| No data at time point | 57 (41.0%) | 77 (21.5%) |  |
| Motor deficits at 3 months post-treatment |  |  | <.0001 |
| No deficits | 46 (33.1%) | 241 (67.1%) |  |
| Weakness | 25 (18.0%) | 35 (9.8%) |  |
| Myelopathy | 3 (2.2%) | 2 (0.6%) |  |
| Both weakness and myelopathy | 8 (5.8%) | 3 (0.8%) |  |
| No data at time point | 57 (41.0%) | 78 (21.7%) |  |
